# Upregulation of TET2 and Resistance to DNA Methyltransferase (DNMT) Inhibitors in *DNMT1*-Deleted Cancer Cells

**DOI:** 10.3390/diseases12070163

**Published:** 2024-07-18

**Authors:** Angelo B. A. Laranjeira, Dat Nguyen, Lorraine C. Pelosof, James H. Doroshow, Sherry X. Yang

**Affiliations:** Division of Cancer Treatment and Diagnosis, National Cancer Institute, National Institutes of Health, Bethesda, MD 20892, USA; angelo_albertoni@yahoo.com.br (A.B.A.L.); diananguyena503@yahoo.com (D.N.); lorraine.pelosof@nih.gov (L.C.P.); doroshoj@mail.nih.gov (J.H.D.)

**Keywords:** azacitidine, 5-aza-4′-thio-2′-deoxycytidine, decitabine, DNA methyltransferase inhibitors (DNMTi), TET2, p16^ink4A^, p15^ink4B^

## Abstract

**Simple Summary:**

Ten-eleven-translocation (TET) 2 is implicated in human cancers such as lung, breast, skin, and kidney cancers, T-cell lymphomas, and leukemia. It is unclear whether TET2 is involved in the re-expression of the p16 tumor suppressor, which was partially silenced by *CDKN2A* gene methylation. The effect of *DNA methyltransferase* (*DNMT) 1* gene status on TET2 expression following DNMT inhibitor treatment remains unknown in cancer. We found that deleting the *DNMT1* gene made cancer cells prone to increased TET2 expression and re-expression of p16 after drug treatment. TET2 is involved in the demethylation of the *CDKN2A* gene and activation of p16 expression, and concomitant resistance to DNMT inhibitors when *DNMT1* is obliterated. The long-sought data for the first time revealed a link between TET2 expression and the activation of p16 expression. The findings should have an impact on reactivating tumor suppressors and cancer treatment.

**Abstract:**

Background: Ten-eleven-translocation (TET) 2 is a member of the TET family of proteins (TET1-3). *DNMT1* gene deletion confers resistance to DNA methyltransferase (DNMT) inhibitors in colorectal, breast, and ovarian cancer cells. Currently, the effect of *DNMT1* gene status on TET2 phenotype following DNMT inhibitor treatment is unclear in human malignancies. Methods: Human colorectal carcinoma HCT116 cells (*DNMT^+/+^*) and their isogenic DNMT1 knockout (*DNMT1^–/–^*) counterpart were treated with DNMT inhibitors. Expression of TET2 and tumor suppressor (p16^ink4A^ and p15^ink4B^) proteins were examined by Western blot. Apoptosis and *CDKN2A* promoter demethylation following drug treatment were detected by Annexin-V apoptosis assay and methylation-specific PCR. Results: TET2 expression was robustly increased in *DNMT1^−/−^* cells by 0.5 µM and 5 µM decitabine and azacitidine treatment. Augmentation of TET2 expression was accompanied by re-expression of p16^ink4A^ and p15^ink4B^ proteins and *CDKN2A* promoter demethylation. TET2 upregulation and tumor suppressor re-expression were associated with resistance conferred by *DNMT1* deletion. Treatment with 5-aza-4′-thio-2′-deoxycytidine at a low 0.5 µM dose only upregulated TET2 and reduced *CDKN2A* promoter methylation, and re-expression of p16^ink4A^ in *DNMT1^−/−^* cells. DNMT inhibitors showed minimal effects on TET2 upregulation and re-expression of tumor suppressor proteins in cells with intact *DNMT1*. Conclusions: *DNMT1* gene deletion made cancer cells prone to TET2 upregulation and activation of tumor suppressor expression upon DNMT inhibitor challenge. TET2 augmentation is concomitant with resistance to DNMT inhibitors in a *DNMT1*-deleted state.

## 1. Introduction

Epigenetic dysregulation is frequently linked to cancer initiation and resistance to agents that target the cancer epigenome [1]. The *DNA methyltransferase (DNMT) 1* gene is located on chromosome 19p13.2, and encodes an enzyme that maintains and propagates DNA methylation patterns in proliferating cells [2,3]. The enzyme DNMT1 and other DNMTs function to transfer a methyl group from S-adenyl methionine (SAM) to the 5 position of cytosine to generate C5-methylcytosine (5-mC) in the context of CpG islands, and is implicated in silencing tumor suppressor genes [4,5]. Inhibition of DNMT1 by DNMT inhibitors as a class of drugs that target the epigenetic regulators and other approaches, such as antisense oligonucleotide, reduces the DNA methylation level and reactivates expression of tumor suppressors such as p16^ink4A^ and/or p15^ink4B^ [6,7]. Cytosine analog decitabine (5-aza-2′-deoxycytidine or aza-dCyd or DAC) and azacitidine (5-azacytidine or AZA) are prototypes of DNMT inhibitors (DNMTi), also known as hypomethylating agents [8]. 5-Aza-4′-thio-2′-deoxycytidine (aza-T-dCyd) is a DNMTi that inhibits DNMT1 in experimental models [9,10]. Decitabine and azacitidine covalently bind to DNMT1 upon incorporation into DNA, blocking DNMT function and demethylating CpG sites during DNA replication [11]. In addition, the DNA-DNMT protein adduct formation causes DNA strand breaks and induces DNA damage response [12,13]. It has been proposed that the reactivation of the silenced tumor suppressor genes through the CpG demethylation was an epigenetic mechanism of anticancer activity of DNMT inhibitors. ^2^ Both decitabine and azacitidine as a nucleoside metabolic inhibitor have been approved for and in standard of care of adult patients with myelodysplastic syndromes (MDS), and chronic myelomonocytic leukemia. However, their application in human solid tumors remains in preclinical and clinical trial development.

Ten-eleven-translocation (TET) 2 encoded by this gene (*TET2*; 4q24) is a member of the TET family of proteins (TET1-3) [14]. They are large (~180 to 230 kDa) multidomain enzymes that convert 5-mC to 5-hydroxymethylcytosine (5-hmC) in DNA through their catalytic dioxygenase activity and initiate demethylation at 5-mCpG sites [15]. TET2 is implicated in human malignancies such as lung, breast, skin, and kidney cancers and frequently in angioimmunoblastic and peripheral T-cell lymphomas and several types of leukemia, and a broad spectrum of cancer cell lines [16].

*DNMT1* gene deletion confers resistance to DNMTi, with reduced levels of DNA damage and apoptotic/cytotoxic effects in colorectal, breast, and ovarian cancer cells [10]. Decitabine and azacitidine, but not aza-T-dCyd, demethylated *CDKN2A/CDKN2B* genes at a relatively high dose of 5 µM in *DNMT1^−/−^* and *DNMT1^+/+^* cells (to a greater degree) and activated the expression of p16^INK4A^ and p15^INK4B^ in HCT116 *DNMT1^−/−^* cells. However, no demethylation or activation of p16^INK4A^ and p15^INK4B^ expression was detected by aza-T-dCyd at a 5 µM dose in HCT116 *DNMT1^−/−^* cells. This was ascribed to the fact that the cells with severe DNA damage were mostly killed or impeded incorporation into DNA (due to thio residue) by aza-T-dCyd at a 5 µM dose.

Currently, it is unclear whether TET2 is implicated in the re-expression of p16^ink4A^ and p15^ink4B^ tumor suppressors despite its widely recognized function of site-specific demethylation in mammalian cells and whether aza-T-dCyd demethylates tumor suppressor genes at low doses. In particular, the effect of *DNMT1* status on the TET2 phenotype following DNMTi treatment remains elusive in cancer cells. No data are available on DNMTi anticancer cell activity or resistance to DNMTi in the context of TET2 upregulation, the demethylation of *CDKN2A* or *CDKN2B* genes, and re-expression of p16^INK4A^ or p15^INK4B^. In this study, we investigated whether TET2 plays a role in demethylating the tumor suppressors and activation of their expression. We revealed the relationship between TET2 upregulation, demethylation, and tumor suppressor re-expression, and concomitant resistance to DNMTi in the absence of *DNMT1*.

## 2. Materials and Methods

### 2.1. Cell Lines and Drugs

Human colorectal carcinoma HCT116 cells (DNMT^+/+^; RRID: CVCL0291) and their isogenic DNMT1 knockout (DNMT1^–/–^) counterpart (HD R02-020) were obtained from Horizon Discovery. The HCT116 DNMT1^–/–^ cell line (Δexons3-5/Δexons3-5) was generated by homozygous knockout of *DNMT1* exons 3-5 encoding the PCNA binding domain [17] and cultured in RPMI-1640 supplemented with 10% FBS and 2 mM L-glutamine. Aza-T-dCyd (NSC777586) and decitabine (NSC127716) were obtained from the Developmental Therapeutics Program (RRID:SCR_003057), Division of Cancer Treatment and Diagnosis, National Cancer Institute (Frederick, MD, USA), and azacitidine (A1287) was purchased from Millipore-Sigma, Corp (Rockville, MD, USA).

### 2.2. MTT Assay

The MTT (3-[4,5-dimethylthiazol-2-yl]-2,5-diphenyltetrazolium bromide) assay for measurement of cytotoxicity has been described previously [10]. In brief, cells (0.4 × 10^4^ cells/well) in triplicates were seeded in 96-well plates and treated with increasing concentrations of DNMT inhibitors for 96 h. The enzymatic reduction of MTT to the insoluble MTT formazan is catalyzed by NAD(P)H-dependent cellular oxidoreductase enzymes in viable cells. The 96-well plates were incubated for an additional 4 h at 37 °C after MTT addition and this was followed by incubating them under the same conditions overnight after adding 10% sodium dodecyl sulfate/0.01 M hydrochloric acid solution. The formazan formed was quantified by measuring the absorbance of the dye solution at 590 nm.

### 2.3. Annexin-V Apoptosis Detection

The cells were treated with DNMT inhibitors for 96 h and, after washing, they were labeled with annexin-V-FITC and propidium iodide (PI)-PE (R&D Systems). The labeled cells were analyzed with a FACSCanto II flow cytometer (Becton Dickinson) using the BD FACSDiva^TM^ software (RRID:SCR_001456).

### 2.4. Western Blot

The Western blot method has been described previously [16]. Briefly, 50 µg of protein was electrophoresed on 7.5% or 4-20% SDS-polyacrylamide gels (Bio-Rad, Hercules, CA, USA). The TET2 polyclonal antibody (Cat# ABE364) was purchased from EMD Millipore and used at a 1 to 2000 dilution for blotting. The other primary antibodies used for the Western blot analysis were CDKN2A/p16^ink4A^ (RRID:AB_776945; Cat# ab40803/clone EP435Y; Abcam), CDKN2B/p15^ink4B^ (Cat# CF505201/clone OT12D11; ThermoFisher Scientific), γH2AX (RRID:AB_309864; Cat# 05-636/clone JBW301; Millipore Sigma), and β-actin (RRID:AB_476692; Cat# A1978/Clone AC-15; Sigma Aldrich), and used in ratios of 1:1000, 1:1000, 1:1000, and 1:30,000, respectively.

### 2.5. Real-Time Methylation-Specific PCR

Methylation-specific PCR (MSP) was performed as previously described by Herman and colleagues [18]. DNA (500 ng) was subjected to bisulphite conversion of cytosine to thymine in DNA using the EpiTect Bisulfite kit (Qiagen). Real-time PCR, with the Syber Green PCR Master Mix as the intercalating dye, was performed using 7900HT Applied Biosystems (RRID:SCR_018060). The reaction mixture in a final volume of 15 µL consisted of 25 ng of bisulphite-modified DNA and 200 nmol/l of each primer. The relative levels of methylated DNA (%) in each sample were calculated according to the equation: Cmeth = 100/[1 + 2(CtCG − CtTG)]% [19]. CtCG represents the threshold cycle for the methylated CG reaction, and CtTG represents the threshold cycle of the unmethylated reaction. The CpGenome universal methylated and unmethylated DNA (Millipore Sigma) were utilized as positive and negative controls. The primer sets near the transcriptional start site used to amplify methylated and unmethylated *CDKN2A* gene have been described previously [18]. Methylated DNA was amplified with *CDKN2A*/p16 gene-specific primers 5′-TTATTAGAGGGTGGGGCGGATCGC-3′ (sense) and 5′-GACCCCGAACCGCGACCGTAA-3 (antisense). The bisulfite-modified DNA was amplified with the specific primers 5′-TTATTAGAGGGTGGGGTGGATTGT-3′ (sense) and 5′-CAACCCCAAACCACAACCATAA-3′ (antisense).

## 3. Results

### 3.1. TET2 Upregulation and Tumor Suppressor Re-Expression by DNMT Inhibitors in DNMT1^−/−^ Cells

**Figure 1:**
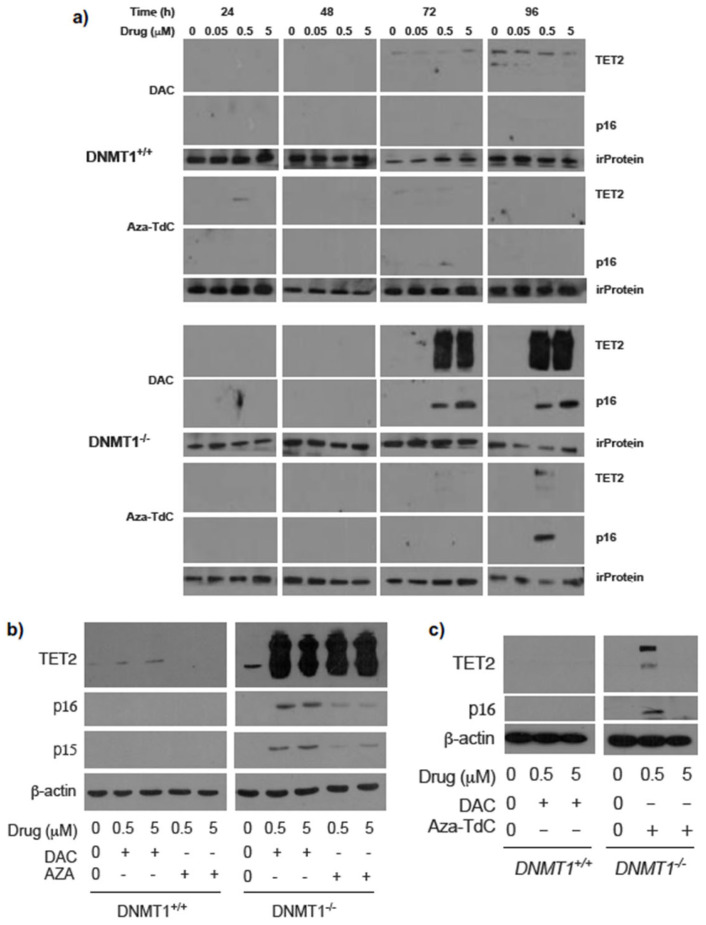
Expression of TET2 and tumor suppressor proteins after DNMTi treatment in HCT116 DNMT1^+/+^ and DNMT1^–/–^ cells. (**a**) TET2 upregulation and p16^ink4A^ re-expression upon exposure to decitabine and aza-T-dCyd for 72h and 96 h in DNMT1^–/–^ cells. irProtein, irrelevant protein. (**b**) TET2 upregulation and p16^ink4A^ and p15^ink4B^ re-expression after decitabine and azacitidine treatment for 72 h. (**c**) Confirmation of TET2 upregulation and p16^ink4A^ re-expression upon exposure to aza-T-dCyd for 72 h in DNMT1^–/–^ versus DNMT1^+/+^ cells. Aza-TdC, aza-T-dCyd; DAC, decitabine; AZA, azacitidine.

To investigate the effect of *DNMT1* gene status on TET2 phenotype following DNMTi treatment and activation of tumor suppressor genes, we examined TET2 expression in *DNMT1* parental and knockout cells. TET2 expression was robustly upregulated after treatment with 0.5 µM and 5 µM decitabine for 72 h and 96 h in *DNMT1^−/−^* cells versus *DNMT1^+/+^* cells (Figure 1a and Supplementary Figure S1a). The augmentation in TET2 expression was accompanied by a dose-dependent re-expression of p16^ink4A^. To confirm the findings, we used another widely used DNMTi, azacitidine, to treat the cells. TET2 expression was markedly increased by azacitidine similarly (Figure 1b and Supplementary Figure S1b). In parallel, both p16^ink4A^ and p15^ink4B^ expressions were reactivated by azacitidine and decitabine. Exposure to aza-T-dCyd upregulated TET2 and re-expressed p16^ink4A^ by 0.5 µM but not by 5 µM treatment for 96 h and 72 h in *DNMT1^−/−^* cells (Figure 1a,c and Supplementary Figure S1c). The data demonstrate a linkage between TET2 augmentation and activation of the expression of tumor suppressor proteins after DNMTi exposure in *DNMT1^−/−^* cells relative to *DNMT1^+/+^* cells.

### 3.2. CDKN2A Promoter Demethylation by DNMTi in HCT116 DNMT1^+/+^ and DNMT1^–/–^ Cells

**Figure 2:**
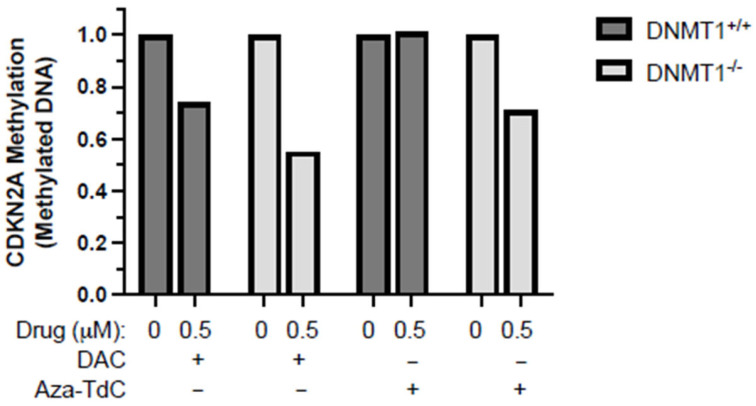
*CDKN2A* promoter demethylation by DNMTi in HCT116 *DNMT1^+/+^* and *DNMT1^–/–^* cells. Shown here is the methylated *CDKN2A* promoter level after exposure to 0.5 μM of decitabine and aza-T-dCyd, as indicated, for 72 h relative to the control. DAC, decitabine. The samples were assayed in triplicate, with at least three independent experiments, and the data above are shown as the ratio of the average methylated DNA in the drug-treated sample compared to the control in a representative experiment.

As demonstrated previously, no demethylation of tumor suppressor genes was detected at a 5 µM dose of aza-T-dCyd, although it is a potent DNMTi in vitro and in vivo [9,10]. We, here, focused on investigating whether the novel DNMTi had a role in demethylation of tumor suppressor genes at a low 0.5 µM dose, along with decitabine as a reference. Aza-T-dCyd demethylated the *CDKN2A* promoter after 72 h of treatment in *DNMT1^−/−^* but not *DNMT1^+/+^* cells (Figure 2). Decitabine demethylated *CDKN2A* to a greater extent in *DNMT1^−/−^* than *DNMT1^+/+^* cells. These data suggest that low-dose aza-T-dCyd demethylated the tumor suppressor promoter following TET2 upregulation in *DNMT1^−/−^* as opposed to *DNMT^+/+^* cells.

### 3.3. DNA Damage, Apoptosis, and Cytotoxic Effects of DNMTi on HCT116 DNMT1^+/+^ and DNMT1^–/–^ Cells

**Figure 3:**
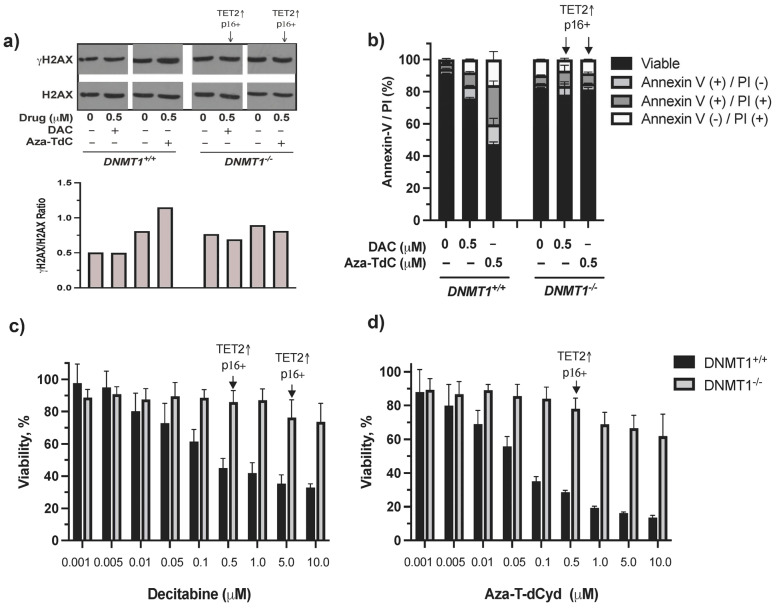
DNA damage, apoptosis, and cytotoxic effects of DNMTi on HCT116 *DNMT1^+/+^* and *DNMT1^–/–^* cells. (**a**) **γ**H2AX expression and quantitation after treatment with decitabine and aza-T-dCyd for 24 h. (**b**) Apoptosis induced by 0.5 μM DNMTi after treatment for 96 h. (**c**) Cytotoxic profiles produced by decitabine. (**d**) Cytotoxic profiles generated by aza-T-dCyd. Arrows indicate the doses that induced TET2 and p16^ink4A^ and/or p15^ink4B^ in *DNMT1^–/–^* cells. DAC, decitabine.

There was minimal induction of DNA damage, cell cycle arrest, and apoptosis by decitabine and azacitidine as well as by aza-T-dCyd at both low and high doses in *DNMT1^−/−^* cells previously [10]. In this study, we aimed to dissect the mechanism of resistance to DNMTi in the context of *DNMT1* gene deletion, TET2 upregulation, and activation of tumor suppressor expression primarily using the low 0.5 µM dose DNMTi. **γ**H2AX and apoptosis were induced by aza-T-dCyd and decitabine (**γ**H2AX was barely detectable by 0.5 µM decitabine) in *DNMT1^+/+^* cells, whereas few such effects were observed in *DNMT1^−/−^* cells despite TET2 upregulation and p16^ink4A^ and/or p15^ink4B^ re-expression (Figure 3a, Supplementary Figure S2a,b). The difference in cytotoxicity between *DNMT1^−/−^* and *DNMT1^+/+^* cells became larger as the drug concentrations increased (Figure 3c,d). Induction of TET2 and tumor suppressor proteins at doses of 0.5 µM and 5 µM by decitabine and 0.5 µM by aza-T-dCyd did not appear to notably alter the course of cytotoxic profiles in *DNMT1^−/−^* cells. Taken together, TET2 upregulation and activation of tumor suppressor proteins are likely a result of riding with the absence of *DNMT1,* which facilitated their upregulation and activation by DNMTi. As such, their induction had a minimal effect on reversing the resistance conferred by *DNMT1* gene deletion.

## 4. Discussion

**Figure 4:**
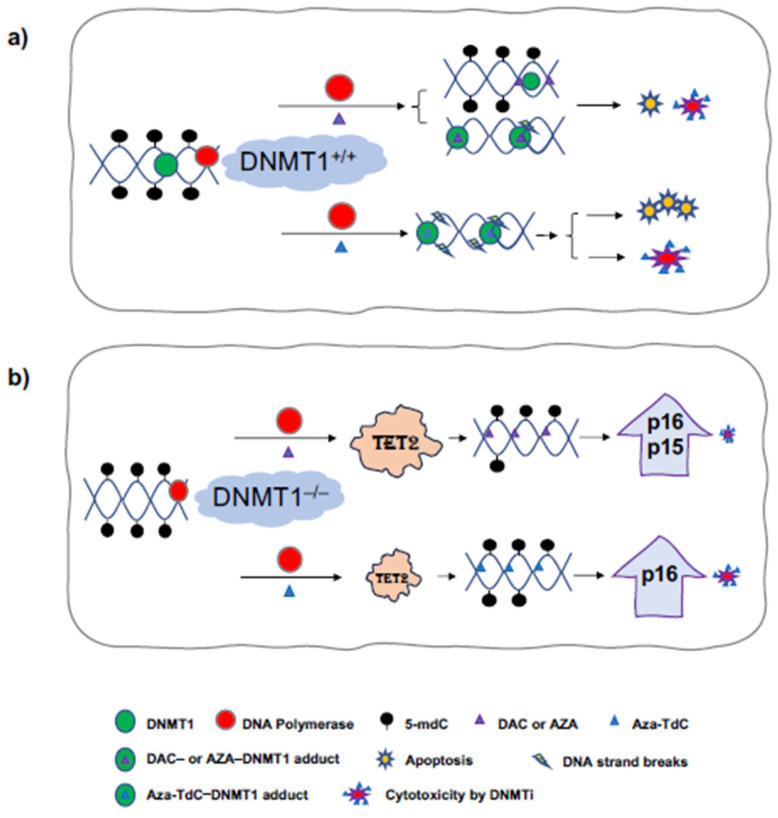
Diagram of DNA damage, TET2 upregulation, demethylation, and activation of tumor suppressor expression by the mechanism of DNMTi anticancer activity using 0.5 μM low-dose DNMTi. (**a**) In DNMT1^+/+^ cells, the incorporation of DAC or AZA into DNA inhibits DNMT1 function and causes DNA demethylation upon replication, and DAC- or AZA-DNMT1 adducts generate little DNA strand breaks, and some degree of cytotoxicity and apoptosis. Aza-T-dCyd produces a greater degree of DNA damage, leading to more apoptosis and cytotoxicity. (**b**) In DNMT1^–/–^ cells, DAC or AZA robustly upregulate TET2 and cause demethylation and re-expression of tumor suppressors to a greater extent, with a minimal cytotoxic effect. Aza-T-dCyd upregulates TET2, leading to demethylation and re-expression of tumor suppressor, and generates some degree of cytotoxicity. Aza-TdC, Aza-T-dCyd; AZA, azacitidine; DAC, decitabine.

Epigenetic-targeted agents including DNMT inhibitors have shown clinical activity in hematological malignancies and are undergoing preclinical and clinical development in human solid tumors [1]. It is imperative to understand the mechanisms of their anticancer activities and resistance surrounding the key regulators of DNA methylation and demethylation, e.g., DNMTs and TET family members. *DNMT1* gene deletion confers resistance to DNMTi in colorectal, breast cancer, and ovarian cancer cells using the gene knockout approach [10]. In this study, we revealed DNA damage, TET2 upregulation, demethylation, and re-expression of tumor suppressors on the underlying mechanism of DNMTi anticancer activity and resistance, particularly in the context of a lower dose (0.5 µM) of DNMTi, in the presence and absence of the *DNMT1* gene (Figure 4). In *DNMT1*^+/+^ cells, decitabine produces little or no detectable DNA damage by **γ**H2AX, some degrees of cytotoxicity, and apoptosis, while aza-T-dCyd generates the DNA strand breaks and greater degree of apoptosis and cytotoxicity. It is noteworthy that 0.5 µM aza-T-dCyd probably continues having more effect on DNA damage than its incorporation into DNA, compared to decitabine, to generate detectable DNA demethylation in *DNMT^+/+^* cells. In *DNMT1^–/–^* cells, decitabine robustly upregulates TET2, with re-expression of tumor suppressors at 0.5 µM and 5 µM doses and aza-T-dCyd at a 0.5 µM dose only upregulates TET2 and induces p16^ink4A^ re-expression. Overall, augmentation of TET2 and activation of tumor suppressors had minimal effects on altering the resistance to DNMTi conferred by *DNMT1* gene deletion.

It has been shown that DNMT1 binds to the *TET2* promoter CpG islands that elevated the DNA methylation levels, leading to the repression or low-level expression of TET2 [19,20]. The deletion of *DNMT1* may have released its repression on the TET2 promoter in the cancer cells and prime these cells for TET2 upregulation upon exposure to DNMTi. After the suppression release by the deletion of *DNMT1*, TET2 expression could have been compensated for an increase through the incorporation of cytosine analogs into the DNA. The more they were incorporated, the greater the degree of the expression of TET2, which was upregulated under circumstances of limited DNA damage instigated by decitabine, azacitidine, and low-dose aza-T-dCyd.

## 5. Conclusions

We revealed the relationship between TET2 upregulation, demethylation and tumor suppressor re-expression, and concomitant resistance to DNMTi in the absence of the *DNMT1* gene. Its deletion made the cancer cells prone to TET2 upregulation and activation of tumor suppressor expression upon DNMT inhibitor challenge. TET2 augmentation contributed to the *CDKN2A* promoter demethylation, tumor suppressor re-expression, and concomitant resistance to DNMT inhibitors in the *DNMT1* gene deleted state.

## Data Availability

The primary data of this study are available from the corresponding author upon request.

## Supplementary Materials

The following supporting information can be downloaded at: https://www.mdpi.com/article/10.3390/diseases12070163/s1, Figure S1: Original Western blot films of Figure 1; Figure S2: Original Western blot films of Figure 2a.
