# Supplementary material for: Upregulation of TET2 and Resistance to DNA Methyltransferase (DNMT) Inhibitors in DNMT1-Deleted Cancer Cells
Source: Diseases. 2024 Jul 18;12(7):163. doi: 10.3390/diseases12070163 (PMC11276550; doi:10.3390/diseases12070163)
Supplement: Supplementary file 1 [file diseases-12-00163-s001.zip › diseases-2992320-supplementary.pdf]

a-a)

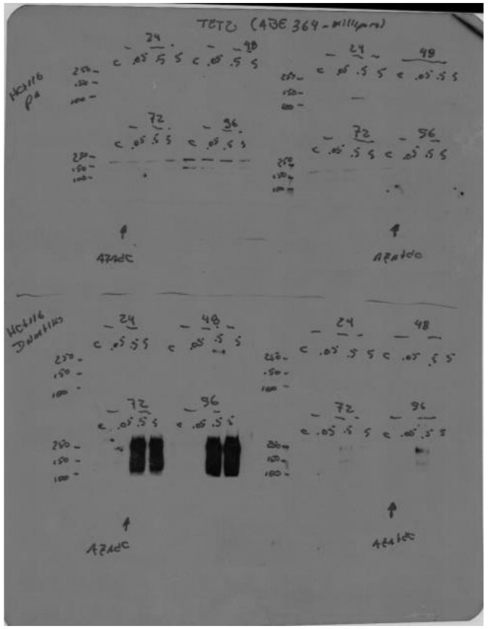

a-b)

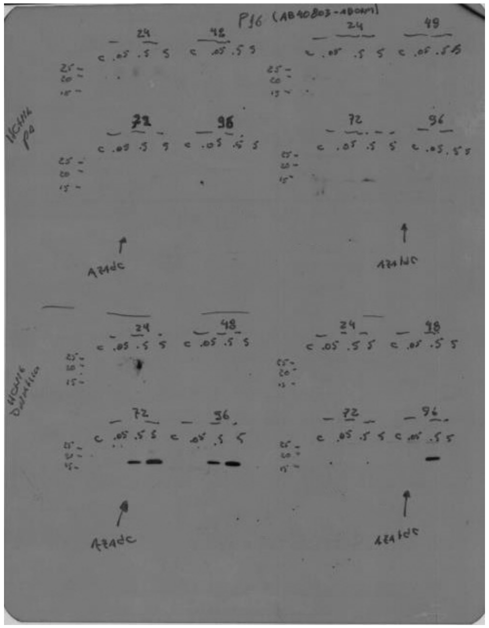

a-c)

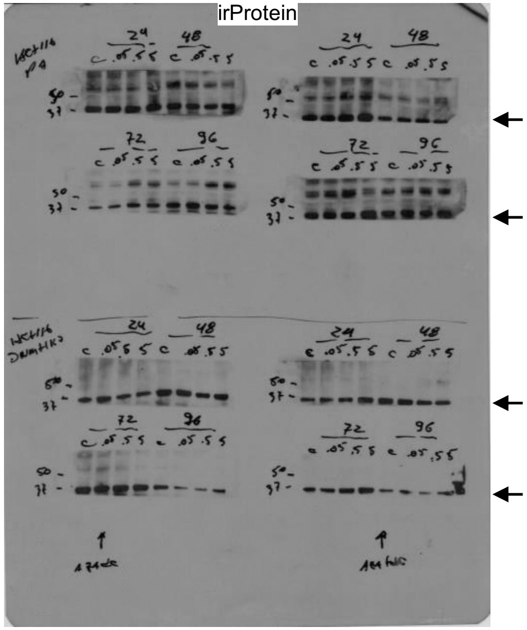

Supplementary Figure 1a. Original Western blot films of Figure 1a. (a-a) TET2, and (a-b) p16^{ink4A} re-expression by drug treatment, and (a-c) irProtein, irrelevant protein. HCT116 pa, HCT116 DNMT1$^{+/+}$; HCT116 DNMT1KO, HCT116 DNMT1$^{-/-}$; Azadc, 5-aza-2′-deoxycytidine; Azatdc, 5-Aza-4′-thio-2′-deoxycytidine.

b-a) b-b) b-c) b-d)

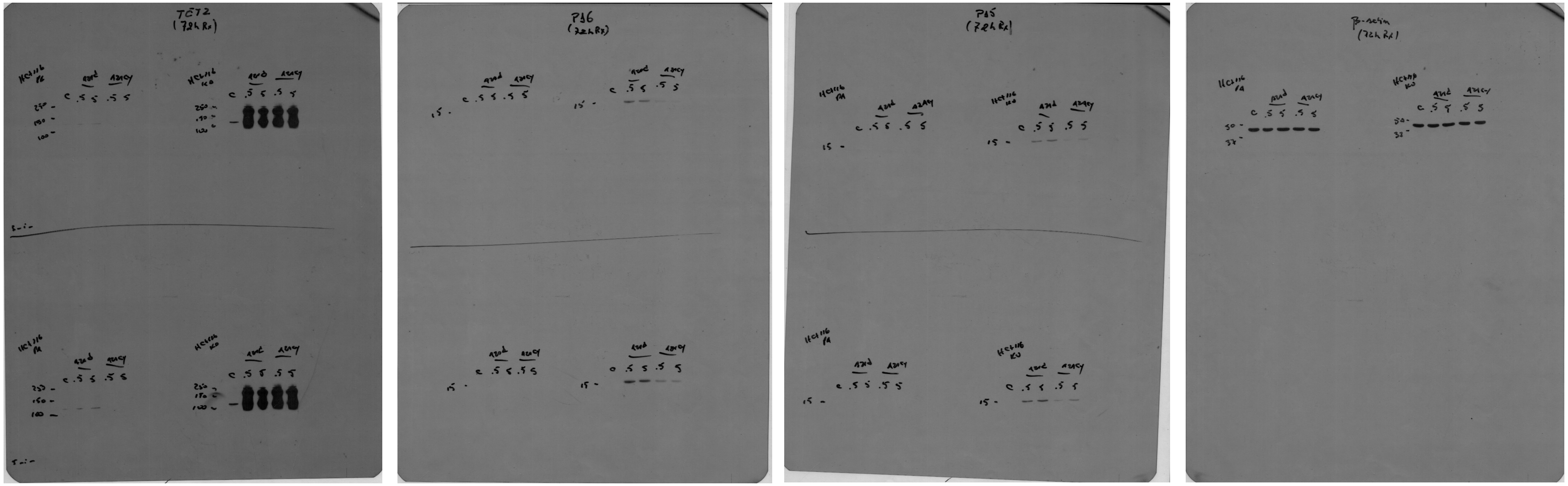

Supplementary Figure 1b. Original Western blot films of Figure 1b. (b-a) TET2, (b-b) $p16^{ink4A}$, and (b-c) $p15^{ink4B}$ re-expression after decitabine and azacitidine treatment for 72 h, and (b-d) β-actin as loading controls. HCT116 pa, HCT116 $DNMT1^{+/+}$; HCT116 DNMT1KO, HCT116 $DNMT1^{-/-}$; Azadc, 5-aza-2′-deoxycytidine; Azacy, 5-azacytidine.

c-a)

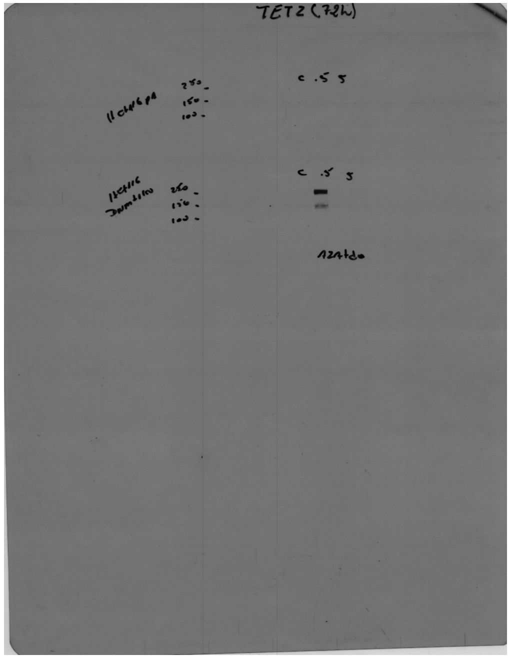

c-b)

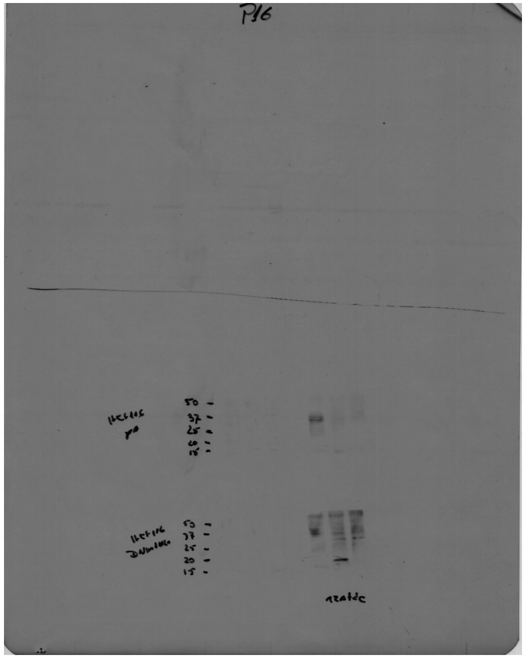

c-c)

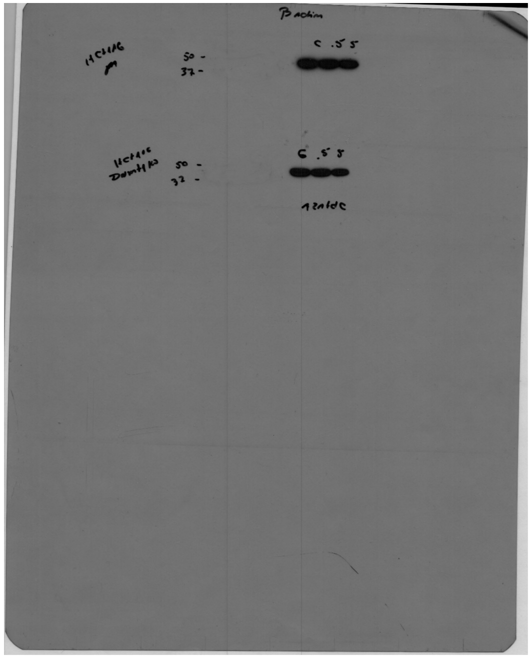

Supplementary Figure 1c. Original Western blot films of Figure 1c. (c-a) TET2 upregulation and (c-b) $p16^{ink4A}$ reexpression after aza-T-dCyd treatment for 72 h, and (c-c) β-actin as loading controls. HCT116 pa, HCT116 $DNMT1^{+/+}$; HCT116 DNMT1KO, HCT116 $DNMT1^{-/-}$; Azatdc, 5-Aza-4′-thio-2′-deoxycytidine.

a-a)

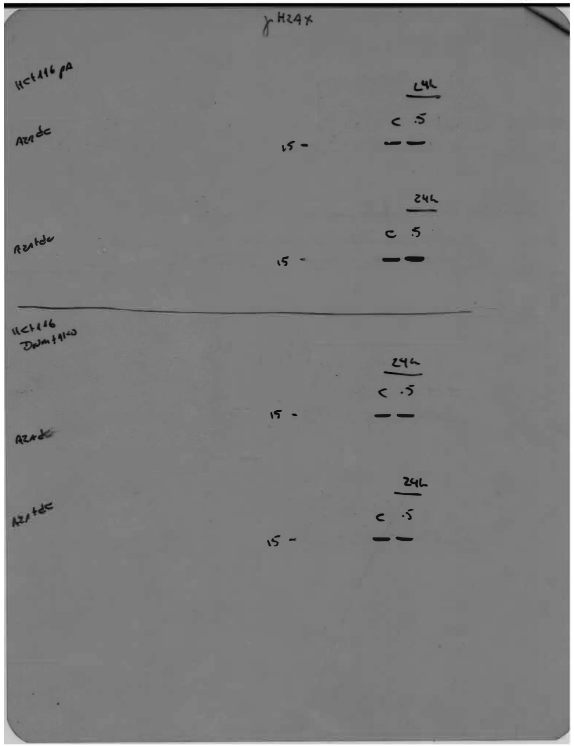

a-b)

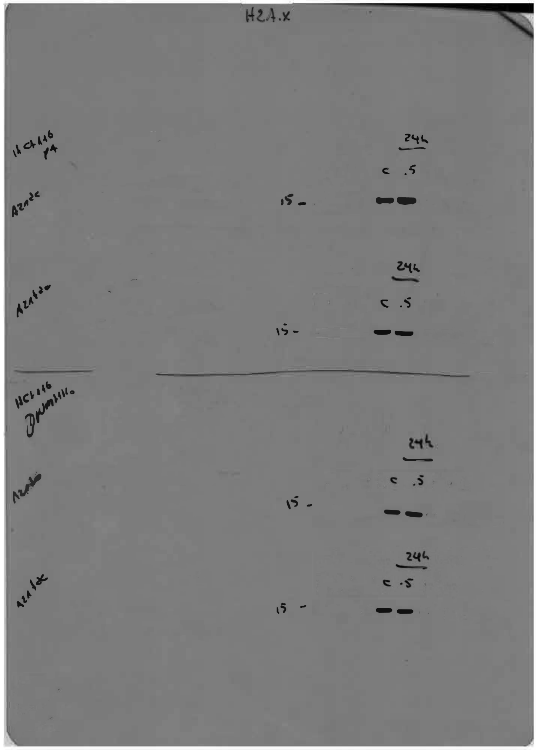

Supplementary Figure 2a. Original Western blot films of Figure 3a. (a-a) **γ**H2AX after drug treatment and (a-b) H2AX as loading controls. HCT116 pa, HCT116 DNMT1$^{+/+}$; HCT116 DNMT1KO, HCT116 DNMT1$^{-/-}$; Azadc, 5-aza-2′-deoxycytidine; Azatdc, 5-Aza-4′-thio-2′-deoxycytidine.
